# Impact of Air Pollution on Metabolic Dysfunction-Associated Fatty Liver Disease

**DOI:** 10.3390/ijms27125168

**Published:** 2026-06-07

**Authors:** Duoduo Lv, Heyu Tang, Lingyun Zhou

**Affiliations:** 1 Center of Infectious Diseases, West China Hospital of Sichuan University, No. 37 GuoXue Alley, Chengdu 610041, China; lvduoduo@scu.edu.cn; 2 Division of Cellular and Molecular Therapy, Department of Pediatrics, University of Florida, Gainesville, FL 32611, USA; heyu.tang@ufl.edu

**Keywords:** air pollution, MAFLD, particulate matter, inflammation, oxidative stress

## Abstract

Metabolic dysfunction-associated fatty liver disease (MAFLD) is now recognized as a leading form of chronic liver disease globally and is strongly associated with metabolic abnormalities. Traditionally, the pathogenesis of MAFLD has mainly been attributed to genetic susceptibility and unhealthy lifestyles (such as high-calorie diets and sedentary behavior). However, in recent years, environmental factors, especially air pollution, have been confirmed as independent risk factors and important promoting factors for MAFLD development and further disease progression. This review summarizes current epidemiological findings on the link between air pollution exposure and MAFLD, while exploring its potential biological mechanisms involving systemic inflammation, oxidative stress, immune alteration, genetic risk, and epigenetic regulation underlying the relationship between air pollution and hepatic steatosis. It also reviews the additive interaction between air pollution and lifestyle or socioeconomic factors in MAFLD. Finally, we also discuss multilevel strategies spanning individual-, community-, national-, and global-level cooperation to address the increasing public health burden caused by air pollution. Therefore, incorporating the assessment and control of air pollution into the comprehensive strategies for MAFLD prevention and treatment has important scientific value and public health significance.

## 1. Introduction

Metabolic dysfunction-associated fatty liver disease (MAFLD) is a complex chronic liver disorder closely associated with obesity, insulin resistance, hypertension, and dyslipidemia, as demonstrated by epidemiological and clinical evidence. Accordingly, MAFLD is commonly regarded as the hepatic manifestation of metabolic syndrome [1]. MAFLD, as an updated term for NAFLD, more accurately captures hepatic steatosis in the context of metabolic dysfunction [2]; therefore, the term MAFLD is used throughout this article. MAFLD has emerged as the leading cause of chronic liver disease worldwide [3]; the global prevalence of MAFLD is estimated to be approximately 25% [4]. Patients with MAFLD commonly present with metabolic dysregulation driven by obesity and insulin resistance, and MAFLD-related mortality is expected to increase alongside the rising burden of type 2 diabetes and cardiovascular disease [5,6]. Genetic susceptibility and epigenetic alterations can also contribute to MAFLD pathogenesis and its secondary diseases [7]. Furthermore, an increasing amount of evidence supports that the gut microbiota has a significant impact on MAFLD [8].

Although the aforementioned factors have been central to MAFLD research, accumulating evidence indicates that environmental factors, especially exposure to air pollutants, may also play a significant role in the etiology of MAFLD [9]. The World Health Organization (WHO) has reported that approximately nine million deaths annually are attributable to environmental pollution [10]. Among the various forms of pollution, air pollution constitutes a major threat to public health and is largely characterized by elevated atmospheric levels of particulate matter (PM), carbon monoxide (CO), nitrogen oxides (NO_x_), and other gaseous pollutants [11]. Notably, exposure to air pollutants may contribute to chronic disease development, with metabolic disorders being among the affected conditions [11].

MAFLD is widely viewed as the hepatic phenotype of metabolic syndrome, with insulin resistance and dysregulation of lipid metabolism constituting its core pathophysiological mechanisms [12]. Current evidence suggests that oxidative stress and inflammatory responses may serve as key pathways linking air pollutant exposure to impaired hepatic lipid metabolism, thereby contributing to the pathogenesis of MAFLD [13]. Although there is extensive evidence linking air pollution to respiratory and cardiovascular diseases [14,15], its involvement in MAFLD remains an emerging field of research. Among the various pollutants of concern, air pollutants have been widely investigated because of their broad monitoring range and the detailed records of their adverse effects on MAFLD [16,17,18]. They also may exert particularly harmful effects because it carries a wide variety of toxic components in PM [19]. Thus, this review summarizes the detailed current evidence regarding the potential contribution of air pollutant exposure to MAFLD progression.

## 2. Air Pollution

**Figure 1:**
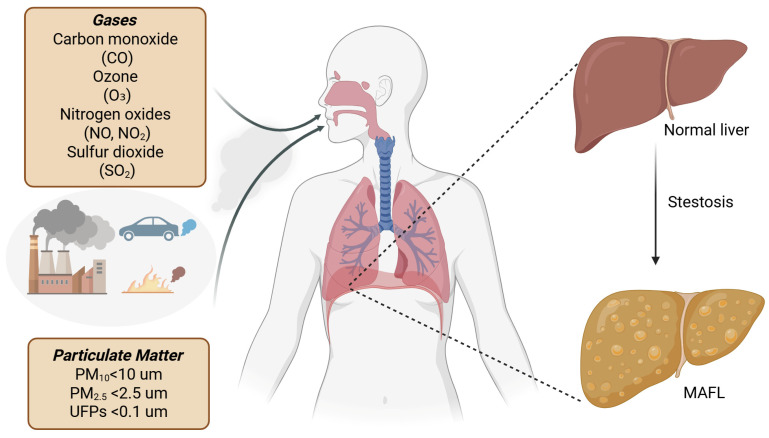
The impact of air pollutants on MAFLD. Air pollutants, including CO, NO_x_, O_3_, SO_2_, PM_10_, PM_2.5_, and UFPs, enter the respiratory tract through inhalation. Fine particles and UFPs may cross the alveolar barrier, and enter the bloodstream, representing a potential pathway linking air pollution exposure to MAFLD. CO, carbon monoxide; O_3_, ozone; MAFLD, metabolic dysfunction-associated fatty liver disease; MAFL, metabolic dysfunction-associated fatty liver; NO_x_, nitrogen oxides; SO_2_, sulfur dioxide; UFPs, ultrafine particles. Created in BioRender. Zhou, L. (2026) https://BioRender.com/hbj4ds3 (accessed on 27 May 2026).

Around the world, millions of people are chronically exposed to air pollutant concentrations that exceed safety standards [20]. At present, there are several air pollution factors with well-established contributions to MAFLD (Figure 1).

### 2.1. Particulate Matter

PM refers to the mixture of solid and liquid particles suspended in the atmosphere, which originates from diverse sources (e.g., agricultural activities, industrial processes, wood and fossil fuels combustion, construction or demolition work, windblown dust, and wildfires) and varies in chemical composition and particle size [21]. PM is classified into the following categories based on particle size: PM_10_ (aerodynamic diameter < 10 μm), PM_2.5_ (fine particles, <2.5 μm), and ultrafine particles (UFPs; <0.1 μm). PM_10_ can deposit in the nasal cavity and upper respiratory tract during nasal breathing and can also enter the lungs. PM_2.5_ and UFPs can penetrate deeper into the alveolar region and enter the bloodstream through alveolar capillaries, inducing systemic inflammatory responses, thereby participating in the initiation and progression of multiple diseases [22,23]. Evidence from epidemiological investigations and animal studies suggest that PM significantly impacts the liver [24,25]. Additionally, some clinical evidence indicates that exposure to PM_2.5_ and PM_10_ may be linked to an increased prevalence of MAFLD [26,27].

### 2.2. CO

CO is a colorless, odorless, toxic gas. Ambient CO is primarily generated by incomplete fossil fuel combustion from vehicle emissions, while contributions from natural sources are generally negligible [28]. A multi-country time-series analysis has shown that even when the ambient CO concentration is below the current air quality guideline threshold, it may still have adverse effects on public health. Short-term exposure to CO has been associated with increased daily mortality [29]. Clinical research also indicates that CO is closely associated with MAFLD in both the general population and individuals with overweight or obesity [30].

### 2.3. O_3_

O_3_ is a major secondary air pollutant generated mainly through photochemical reactions involving volatile organic compounds and NO_x_ [31]. Alongside global warming, the rapid industrialization and urbanization around the world have significantly increased the global ambient O_3_ concentrations. The Global Burden of Disease 2019 study reported that ambient O_3_ contributed to an estimated 365,000 deaths worldwide, including 93,200 in China [32]. Mechanically, O_3_ is a strong oxidant gas that may cause oxidative damage to human cells and tissues, which can trigger immune–inflammatory responses in the lungs [33]. These inflammatory responses may “spill over” into the circulatory system, increasing the risk of multiple organ diseases by affecting systemic inflammation and causing endothelial dysfunction [34].

### 2.4. NO_x_

NO_x_, primarily comprising nitric oxide (NO) and nitrogen dioxide (NO_2_), acts as a key precursor in atmospheric photochemical reactions, promoting the formation of O_3_ and secondary inorganic aerosols [35]. Previous studies have shown that long-term exposure to NO_X_ and NO_2_ is associated with elevated mortality risk from hypertension, ischemic heart disease, and heart failure [36]. In addition, long-term exposure to NO_2_ is associated with elevated MAFLD risk [37].

### 2.5. SO_2_

Sulfur dioxide (SO_2_) is a typical gaseous air pollutant that is derived from the combustion process of fossil fuels and industrial production activities. Anthropogenic activities continuously raise atmospheric SO_2_ levels, posing persistent threats to human systemic health. Epidemiological investigations have confirmed that chronic SO_2_ exposure has a positive relationship with MAFLD risk [38].

### 2.6. Other Adsorbed Compounds

As mentioned previously, PM carries diverse toxic compounds (including polycyclic aromatic hydrocarbons) and carbon particles (e.g., transition metals and lipopolysaccharides), which vary depending on the PM source [39]. These adsorbed compounds can cause inflammatory liver injury, as evidenced by serum biochemical and histopathological analyses [40].

## 3. Effect of Air Pollution on MAFLD

Numerous epidemiologic and experimental studies indicate that short- and long-term exposure to ambient air pollution may increase the risk of MAFLD (Table 1).

### 3.1. Short-Term Effects

Several epidemiologic studies have suggested that short-term exposure to high PM levels is directly linked to increased morbidity in MAFLD patients. Air pollutant exposure is associated with the number of outpatient visits for MAFLD in China: a 10 µg/m^3^ increase in PM (PM_10_ and PM_2.5_) and NO_2_ concentrations corresponded with 0.82 and 0.86% increases in MAFLD outpatient visits [57]. PM_2.5_ exposure is typically lower in the USA than in China, which may reflect differences in environmental pollution intensity, behavioral exposure patterns, and susceptibility across populations. Moreover, available evidence indicates that exposure to ambient PM may increase MAFLD morbidity, even under low-level exposure conditions.

### 3.2. Long-Term Effects

Long-term exposure to PM_2.5_ is significantly associated with an elevated prevalence and higher incidence of MAFLD in a dose-dependent manner [58]. For example, a cross-sectional study including 90,086 Chinese adults found that higher PM_2.5_ exposure was associated with greater odds of MAFLD [59]. Hyun-Jin Kim et al. also reported a positive association between PM_2.5_ and serum gamma glutamyl transferase levels [60]. Evidence from long-term PM_2.5_ exposure studies suggests that it may promote hepatic triglyceride accumulation and markedly elevate cholesterol levels, trigger lipid toxicity, and cause metabolic dysfunction [61]. Similar effects were observed with other pollutants such as NO_x_, O_3_, and CO, suggesting that air pollution has potential hepatotoxic effects [62]. A United Kingdom cohort study on long-term air pollution exposure further found that elevated levels of several air pollutants were linked to a higher prevalence of MAFLD [63].

Another European study showed that long-term exposure to ambient air pollution, even at relatively low concentrations, may be related to multiple key health endpoints [64]. As a major advanced chronic liver disease resulting from MAFLD, cirrhosis may also be associated with air pollution [65]. Consistent findings was also reported in a cohort study from Hong Kong, which showed that each 1 μg/m^3^ increase in average hourly PM_2.5_ concentration corresponded to increases of 0.21 and 0.37 deaths per 100,000 population from chronic liver disease and cirrhosis, respectively [66]. Furthermore, studies have indicated that individuals with existing metabolic disturbances such as obesity and insulin resistance [13,67,68], as well as those carrying specific genetic risk variants like *PNPLA3* [69], may be more susceptible to the hepatotoxic effects of air pollution. Despite challenges related to residual confounding and individual exposure assessment, the high degree of consistency across multiple levels of evidence strongly indicates that air pollution is a significant and non-negligible environmental risk factor in the development and progression of MAFLD.

## 4. Pathophysiology of Air Pollution-Induced MAFLD

### 4.1. Systemic Inflammation and Oxidative Stress

**Figure 2:**
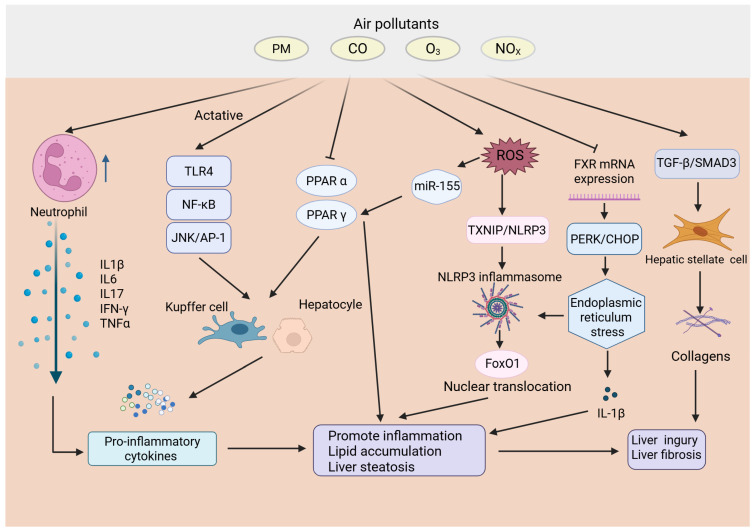
Air pollutants induce systemic inflammation and oxidative stress, which could lead to MAFLD. After exposure to air pollutants, the number of inflammatory cells increases and inflammatory signaling pathways (including the TLR4, NF-κB, and ROS/miR-155/PPARγ pathways) are upregulated, releasing a large amount of pro-inflammatory factors. Meanwhile, PPARα and PPARγ are inhibited, ultimately causing inflammatory responses, lipid accumulation, and hepatic steatosis. Air pollutants generate excess ROS and activate the TXNIP/NLRP3/FoxO1 pathway while promoting liver inflammation. Additionally, air pollutants reduce the expression of FXR, thereby triggering endoplasmic reticulum stress and contributing to the development of hepatic steatosis. Air pollutants cause liver damage through the TGF-β signaling pathway, ultimately leading to the progression of MAFLD. FoxO1, forkhead box protein O1; FXR, farnesoid X receptor; NLRP3, NOD-like receptor pyrin domain 3; MAFLD, metabolic dysfunction-associated fatty liver disease; PPAR, peroxisome proliferator-activated receptor; ROS, reactive oxygen species; TXNIP, thioredoxin-interacting protein; TLR4, toll-like receptor 4. Created in BioRender. Zhou, L. (2026) https://BioRender.com/9hax05k (accessed on 27 May 2026).

Reactive oxygen species (ROS) comprise a group of highly reactive oxidizing molecules or ions, including hydroxyl radical, hydrogen peroxide, peroxynitrite, and superoxide anion, which can oxidize proteins, lipids, and DNA [70]. Under physiological conditions, the liver maintains a robust antioxidant defense system that effectively counterbalances endogenous ROS production. However, prolonged exposure to higher levels of air pollutants can impair this protective capacity, resulting in sustained oxidative stress and subsequent hepatocellular damage (Figure 2). Previous studies have found PM_2.5_ can induce oxidative stress and hepatic inflammation, thereby interfering with normal lipid metabolism in the liver of mice [13]. Persistent low-grade inflammation is considered an important mechanism underlying PM_2.5_-related metabolic dysfunction and MAFLD progression. Brook et al. reported that even a 2-h exposure to concentrated PM was sufficient to rapidly increase total white blood cell and neutrophil counts [71]. Another study demonstrated that PM exposure activated hepatic inflammatory signaling (p-p65 and p50) and cytokines in the liver [72]. For example, a cross-sectional study showed that a higher PM_2.5_ concentration was significantly associated with elevated levels of interleukin (IL)-1β, IL-6, IL-17, and tumor necrosis factor alpha (TNFα) [73]. Inhaled pollutants, such as NO_x_, volatile organic compounds and polycyclic aromatic hydrocarbons, have been reported to contribute to impaired liver function in both animal and human studies [74]. Exposure to PM_2.5_ can activate inflammatory signaling cascades mediated by c-Jun N-terminal kinase, nuclear factor κB (NF-κB), and Toll-like receptor 4 [75]. Furthermore, exposure to PM_2.5_ inhibits the expression of peroxisome proliferator-activated receptor (PPAR) γ and PPARα in the liver, thereby disrupting the lipid and glucose balance in Kupffer cells and liver cells, leading to lipid accumulation [76]. PM_2.5_ exposure has been shown to aggravate hepatic lipid deposition in mice fed a high-fat diet, through regulation of the ROS/miR-155/PPARγ signaling pathway [77].

Inflammatory responses and oxidative stress are the key triggers for the development of liver metabolic disorders, an increase in lipid accumulation in the liver, and chronic hepatic inflammation caused by exposure to PM_2.5_; these detrimental effects can be alleviated through suppression of NF-κB signaling and oxidative stress, leading to reduced inflammation and oxidative injury [13]. PM_2.5_ increases the levels of the fatty acid translocase CD36 and promotes lipid accumulation by reducing the expression of miR-26a [48]. PM_2.5_ exposure could also exacerbate MAFLD via the ROS/TXNIP/NLRP3 pathway, which promotes FoxO1 nuclear translocation and hepatic lipid accumulation [78]. Activation of farnesoid X receptor (FXR) suppresses NLRP3 inflammasome activation triggered by endoplasmic reticulum stress in hepatocytes, thereby alleviating liver damage (hepatocyte death, inflammation, and fibrosis) via the PERK–CHOP signaling pathway [79]. Indeed, PM_2.5_ induces lipid accumulation through downregulation of FXR, and promotes collagen deposition by activating TGF-β signaling, resulting in oxidative damage and fibrosis [80,81]. It should be noted that NLRP3 inflammasome activation is not unique to air pollution exposure; rather, it is a common inflammatory response to multiple metabolic signals, with air pollution potentially acting as an environmental amplifier in MAFLD progression. Collectively, these studies suggest that air pollutants may promote hepatic steatosis and MAFLD progression by modulating inflammatory and oxidative stress-related pathways.

### 4.2. Air Pollution Exposure and Immune Alterations

PM is being increasingly recognized as an immunotoxic environmental exposure. The available evidence suggests a mixed association of NO_2_ and a negative association of O_3_ with the CD4^+^/CD8^+^ ratio [82]. Recent studies showed that PM_2.5_ exposure activates Kupffer cells in murine liver tissues, indicating that PM_2.5_ represents a risk factor for MAFLD progression [76,83]. Ruxianguli Aimuzi et al. suggested that PM_2.5_ and NO_2_ exposure was associated with an increased risk of MAFLD, partially due to perturbation of circulating proteins involved in immune response pathways [42]. In high-fat- and high-sucrose-diet mouse models, exposure to air pollution particles increased inflammatory cells involved in innate immunity and raised local inflammatory cytokine levels [84]. In addition, a study has confirmed that CC-chemokine receptor 2 (CCR2), which plays a critical role in the entry of innate immune cells into tissues, also mediates obesity-associated macrophage infiltration into adipose and hepatic tissues [85]. PM_2.5_ mediates insulin resistance by regulating VAT inflammation and hepatic lipid metabolism via both CCR2-dependent and independent pathways [86]. These findings collectively indicate that air pollution-induced increases in innate immune cells and elevation of pro-inflammatory cytokines in the liver contribute to MAFLD progression.

### 4.3. Air Pollution and Genetic Risk

The multiple-hit hypothesis of MAFLD posits that the disease arises and progresses through the synergistic interplay of genetic susceptibility and environmental exposures. Genetic polymorphism at codon 148 of the patatin-like phospholipase domain-containing protein 3 (*PNPLA3*), a gene encoding a lipase, has been implicated in hepatic lipid accumulation, inflammatory injury, and fibrotic progression [87,88]. reported an interaction between air pollutant exposure and the *PNPLA3* I148M genotype in relation to liver fat fraction and multiomics features [44].

Air pollutants may contribute to MAFLD development not only by inducing oxidative stress and inflammatory responses but also through genotoxic mechanisms that cause direct DNA damage. For example, evidence from in vitro, in vivo, and human studies suggests that air pollution exposure may impair mitochondrial respiratory function and alter mtDNA copy number [89]. For example, decreased mtDNA copy numbers were associated with higher ambient PM_10_ exposure [90].

### 4.4. Epigenetic Regulation Mediating the Association Between Air Pollution and Hepatic Steatosis

**Figure 3:**
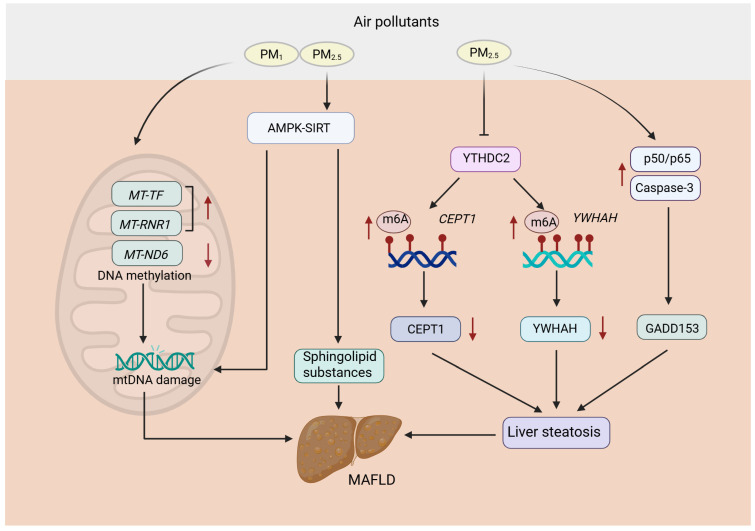
Exposure to different air pollutants induces epigenetic changes, leading to MAFLD. These epigenetic alterations include changes to DNA methylation, which can cause mitochondrial damage and liver steatosis. CEPT1, choline/ethanolamine phosphotransferase 1; MAFLD, metabolic dysfunction-associated fatty liver disease; MT-ND6, mitochondrial-encoded NADH dehydrogenase 6; mtDNA, mitochondrial DNA; MT-TF, mitochondrial transfer RNA for phenylalanine; MT-RNR1, mitochondrial 12S ribosomal RNA; AMPK-SIRT, AMPK-induced sirtuin; YTHDC2, YTH domain-containing 2; YWHAH, tyrosine 3-monooxygenase/tryptophan 5-monooxygenase activation protein eta. Created in BioRender. Zhou, L. (2026) https://BioRender.com/aeubory (accessed on 27 May 2026).

Accumulating fundings indicate that air pollution exposure can induce epigenetic alterations, which may be involved in the development of hepatic steatosis (Figure 3). Environmental exposure has been significantly associated with alterations in gene expression, and air pollution may further affect epigenetic regulation through DNA methylation. For example, there is emerging evidence that suggests that N6-methyladenosine (m6A) modifications play a pivotal role in regulating steatosis, with the m6A-binding protein YTHDC2 suppressing lipid accumulation by modulating the stability of lipogenic gene mRNA [91]. For instance, Yan et al. found that PM_2.5_ downregulated the m6A reader YTHDC2 and increased m6A methylation of *CEPT1* and *YWHAH* transcripts, resulting in reduced mRNA stability and expression of these lipid metabolism-related genes, ultimately promoting hepatic steatosis [92].

PM in ambient air induces mitochondrial damage in the liver [93]. A recent study demonstrated that exposure to PM_2.5_ components caused a significant increase in caspase-3-positive hepatocytes (caspase-3 is an important effector involved in apoptosis), and was accompanied by elevated protein expression of growth arrest and DNA damage-inducible protein 153 [72]. Beyond mitochondrial injury, PM exposure may also affect mitochondrial epigenetic regulation. Altered mtDNA methylation can serve as an indicator of PM exposure and may contribute to lipid metabolism disruptions [94]. A prior study indicated that mitochondrial epigenetic modifications are linked to elevated MAFLD risk, and that hepatic *MT-ND6* methylation status and gene expression are related to disease histological severity [95]. Notably, exposure to PM_1_ has been associated with altered methylation of mitochondrial *MT-TF* and *MT-RNR1* and elevated mtDNA copy number [96]. In addition, recent studies have linked MAFLD to impaired TCA cycle function, a key mitochondrial metabolic pathway located mainly in the mitochondrial matrix [97,98]. Additionally, multi-omics data indicate that PM_2.5_ exposure can lead to the development of MAFLD via the activation of AMPK signaling by PM_2.5_ exposure, which causes mitochondrial damage and the accumulation of sphingolipid substances [99].

## 5. Association Between Exposure to Air Pollutants and MAFLD

### 5.1. Assessment of Air Pollution-Related Histopathological and Functional Alterations in MAFLD

Liver functional impairment can be evaluated through both histopathological and functional indicators. Histologically, progressive liver injury is characterized by hepatocyte swelling or degeneration, hepatocyte death, infiltration of inflammatory cells, bile stasis changes, bile duct reactions, and, in the case of persistent damage, activation of hepatic stellate cells (HSCs), followed by collagen deposition and fibrotic remodeling [100]. Consistent with these pathological mechanisms, animal studies have shown that PM_2.5_ exposure can promote the full spectrum of MAFLD/metabolic dysfunction-associated steatohepatitis (MASH)-like pathology. For example, after PM_2.5_ exposure, mice displayed liver steatosis, inflammatory changes, and fibrosis, as well as reduced glycogen storage and impaired insulin sensitivity [76]. The fibrogenesis induced by exposure to concentrated ambient PM_2.5_ is associated with increased collagen expression, activation of TGF-β signaling, suppression of PPARγ, and collagen production in HSCs, with NADPH oxidase identified as a key mediator of PM_2.5_-induced liver fibrogenesis [80]. These histological changes can also be further confirmed by indicators related to MAFLD progression, including increased expression of extracellular matrix-related genes such as α-SMA and COL1A1 [101]. Functionally, air pollution can cause liver dysfunction, damaging the metabolic, excretory, and synthetic capabilities of hepatocytes [102]. Therefore, in studies assessing the impact of air pollution on the liver, the disease progression should be interpreted by comprehensively evaluating specific histopathological changes and biochemical abnormalities as they collectively reflect the progression from liver cell stress and inflammation related to pollutants to fibrotic remodeling and gradual deterioration of function.

### 5.2. Linking Air Pollutant Exposure and MAFLD-Related Disease Progression

Recently, there has been a considerable increase in the amount of evidence linking air pollutant exposure to MAFLD progression, including MASH, advanced fibrosis, cirrhosis, and hepatocellular carcinoma (HCC) [65,103,104,105]. MASH is histologically defined by hepatic steatosis, hepatocyte ballooning, and lobular inflammation, whereas progressive liver fibrosis results from the excessive deposition of abnormal extracellular matrix within hepatic tissue [106]. PM_2.5_ may promote pro-inflammatory factor expression in adipocytes and activate Kupffer cells, further aggravating hepatic inflammation in MASH [13,107]. Animal studies have verified that increased expression of fibrosis-related factors and collagen accumulation in the livers of air pollution-exposed MAFLD mice contributed to the development of a MASH-like phenotype and liver fibrosis [107,108]. PM_2.5_ promotes Drp1-mediated mitophagy, inducing HSC activation and hepatic fibrosis via regulating miR-411 [109]. In fibrotic liver tissues, transforming growth factor-beta1 upregulates Ndfip1, which facilitates TrkB degradation through the ubiquitin–proteasome pathway and subsequently activates downstream fibrogenic genes [110]. Xabier Unamuno et al. discovered that the NLRP3 inflammasome may drive inflammation in adipose tissue and promote extracellular matrix remodeling, which can accelerate the fibrotic process during the development of MAFLD. PM_2.5_ has been strongly associated with HCC mortality and may potentially contribute to HCC development through carcinogenic components such as metals and polycyclic aromatic hydrocarbons [111,112,113]. Additionally, air pollution may also disturb the gut microbiota, weaken intestinal barrier function, promote endotoxin translocation, and alter bile acid metabolism, resulting in exacerbation of MAFLD [114,115].

### 5.3. Air Pollution-Related Metabolic Dysfunction in MAFLD

Disrupted metabolic homeostasis is a major driver of MAFLD development and progression. Air pollution exposure may aggravate this process by disturbing systemic and hepatic metabolic homeostasis. Long-term exposure to ambient air pollutants was in connection with increased MAFLD risk and severity, particularly among individuals with a higher body mass index and larger waist circumference [116]. In experimental and epidemiological studies, exposure to PM_2.5_ and other air pollutants has been related to insulin resistance [86,117], glucose intolerance [76,118], dyslipidemia [13,119], and increased hepatic lipid accumulation [120]. Pollutant-induced oxidative stress and inflammatory activation may impair insulin signaling in metabolic tissues, while obesity-related inflammation and adipose tissue dysfunction can further increase circulating free fatty acids and pro-inflammatory mediators [75,76]. These changes may enhance hepatic lipid influx, promote de novo lipogenesis, reduce lipid oxidation or export, and ultimately contribute to hepatic steatosis and MAFLD progression. Therefore, air pollution may not only act as an external environmental stressor, but it may also amplify the core metabolic abnormalities underlying MAFLD.

### 5.4. Effects of PM Sources and Composition on MAFLD

The effects of PM on MAFLD may vary according to its chemical composition, particle size, and emission source. Combustion-derived PM, especially traffic-related particles from diesel and gasoline exhaust, often contains elemental carbon, organic carbon, NO_x_, and volatile organic compounds, which may promote hepatic oxidative stress and inflammatory signaling [121]. For example, exposure to low concentrations of traffic-derived PM_2.5_ over an extended period may facilitate pathological changes linked to MAFLD risk [56]. Traffic-related air pollution may exacerbate hepatocellular injury in children with pre-existing MAFLD, as indicated by increased CK-18 levels, a marker of hepatocyte apoptosis [122,123]. Metal-rich PM, which can contain iron, copper, nickel, vanadium, lead, or cadmium, may further enhance reactive oxygen species generation, mitochondrial dysfunction, and hepatocyte injury through redox-active or toxic metal-mediated mechanisms [72,124]. In addition, PM carrying polycyclic aromatic hydrocarbons may activate xenobiotic metabolism and inflammatory pathways, thereby contributing to lipid metabolic disturbances, hepatocellular stress, and potentially carcinogenic progression [124]. These compositional differences may partly explain the heterogeneity of reported associations between PM exposure and MAFLD-related outcomes. Future studies should focus on identifying the signaling pathways underlying the effects of PM exposure on MAFLD.

### 5.5. Susceptibility to Air Pollution-Related MAFLD

Susceptibility should also be considered when interpreting the relationship between air pollution and MAFLD. Current evidence suggests that age and sex may affect the burden of fatty liver disease. For example, MAFLD prevalence was higher among older adults and men, with individuals aged ≥50 years accounting for a relatively large proportion of the MAFLD group than the non-MAFLD group [125]. Yanqi Lan et al. found an association between long-term air pollution exposure and increased susceptibility to liver fibrosis in older patients with MAFLD [105]. Animal experiments have further confirmed that ambient PM_2.5_ exposure inhibited the hypothalamic–pituitary–adrenal axis and suggested sex-associated differences in its effects on insulin resistance and hepatic lipid metabolism [120]. In pediatric populations, air pollution exposure may contribute to hepatic steatosis, but the true burden of pediatric MAFLD remains difficult to define because the current evidence often relies on surrogate markers, which may lead to underestimation or overestimation due to diagnostic limitations and controversies in children and adolescents [126,127]. Future studies should further assess how population-specific factors, such as age and sex, influence air pollution-related MAFLD progression.

## 6. Additive Interaction of Ambient Air Pollution and Lifestyle in MAFLD

Unhealthy lifestyle behaviors (including poor dietary habits and insufficient physical activity) have been strongly linked to MAFLD risk, often interacting with air pollution, which affects fatty liver disease outcomes [128,129]. Xinxin Kong et al. reported that the additive interaction between lifestyle factors and combined air pollutant exposure accounted for 36% of MAFLD risk, suggesting that lifestyle behaviors may modify the association between ambient air pollution and MAFLD risk [129]. Additionally, lower educational attainment and limited financial resources may contribute to unhealthy dietary patterns, as individuals with lower income and education levels are more likely to consume inexpensive, energy-dense foods [130]. Furthermore, individuals with lower educational attainment may have limited awareness of the protective roles of healthy eating and physical activity against chronic diseases, which may reduce their motivation to adopt healthier lifestyle behaviors. This can result in less motivation to incorporate more healthy lifestyle habits.

## 7. Synergistic Effects of Socioeconomic Status and Air Pollution on MAFLD

Many studies in North America have also documented that communities and individuals with a low socioeconomic status are exposed to higher air pollution levels than those with a high socioeconomic status [131,132]. However, observations in the United States may not be directly applicable to China and other regions, as they are grounded in historically, socially, economically, politically, urbanistically, and industrially distinct conditions [133,134,135]. Jiawen Liu et al. suggested that ambient NO_2_ and PM_2.5_ concentrations are higher for individuals with a high vs. low socioeconomic status in China [136]. As income rises, the associated lifestyle modifications contribute to a progressive increase in serum triglyceride concentrations and hepatic fat content [137]; when compounded by long-term exposure to ambient air pollution, this cascade promotes the development of hepatic steatosis [138]. Moreover, populations with a lower socioeconomic status often experience reduced access to structural and environmental resources—such as green spaces, social capital, and high-quality housing—that may buffer or mitigate the adverse health effects associated with elevated air pollution exposure [139]. Additionally, researchers found that individuals with diagnosed MAFLD had socioeconomic factors (education level, marital status, and country of birth) and air pollution exposure levels that were associated with higher rates of prevalent and incident major adverse liver outcomes [140,141]. Overall, available evidence regarding the association between air pollution exposure and MAFLD remains limited in low- and middle-income countries.

## 8. Challenges and Future Directions

MAFLD prevention has increasingly focused on multilevel strategies that address personal behaviors, social contexts, physical environments, and policy measures; however, evidence supporting the effective reduction in combined environmental and social risks remains limited but encouraging.

### 8.1. Epidemiological Evidence: From Association Toward Causal Inference

A substantial body of observational evidence supports the association between air pollution and MAFLD, with the research paradigm evolving from cross-sectional studies using serum biomarkers to prospective cohort designs that strengthen the causal inference [100,104,142]. Despite these advancements, there are still challenges related to residual confounding factors and exposure assessment. Indeed, most research results are limited to correlational studies, which highlights the urgent need for rigorous research. More studies should prioritize natural experiments or intervention studies, longitudinal cohort studies that include repeated exposures and phenotypic characteristics from an early stage of life, and emerging causal inference methods that integrate genetic or exposure group data, to go beyond correlational evidence. It is therefore crucial to emphasize the need for further comprehensive and well-designed studies to more fully elucidate the complex and multifaceted interactions between air pollution exposure and the development and progression of MAFLD.

### 8.2. Comprehensive Approach to Address the Impact of Air Pollution on MAFLD

**Figure 4:**
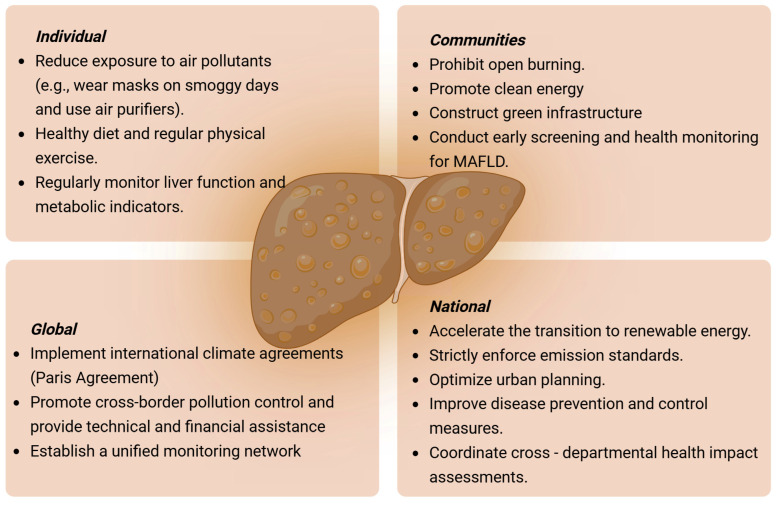
Current or potential air pollution interventions at various intervention levels. Created in BioRender. Zhou, L. (2026) https://BioRender.com/dvlqizg (accessed on 27 May 2026).

Although air pollution is a well-known factor for human health, its recognition as an MAFLD risk factor has been frequently overlooked. Evidence-based and multi-level intervention measures need to be implemented to mitigate air pollution’s adverse effects on the development and progression of MAFLD (Figure 4). Individuals should minimize pollution exposure, adopt healthy lifestyles, and regularly have their liver health checked. As discussed above, unhealthy lifestyle factors may exacerbate the impact of air pollution on MAFLD risk. Individuals, particularly men and those with obesity, should adopt protective lifestyle measures to reduce the adverse health effects of ambient air pollution (e.g., improving their diet, increasing their physical activity, quitting smoking, etc.) [58]. Furthermore, research has confirmed that inadequate and inequitable climate change response measures have made the communities with the least resources more vulnerable to the health impacts of climate change, and more susceptible to the health hazards caused by energy poverty and air pollution generated by fossil fuels [143]. One study found that residential access to green and blue spaces, including natural vegetation areas and water bodies, has been linked to a reduced risk of MAFLD [144]. Thus, communities can adopt a comprehensive intervention strategy that combines emission reduction with health promotion. At the national level, efforts should be made to accelerate the transition from fossil fuels to renewable energy, strictly enforce emission standards for industries and transportation, optimize compact urban planning and clean public transportation, and incorporate air pollution into the prevention and control system for metabolic diseases, as well as improve cross-departmental health impact assessments. Globally, international climate agreements such as the Paris Agreement should be fulfilled, promoting cross-border pollution control and technical and financial assistance, establishing a unified monitoring network, and integrating air pollution prevention into the global action framework for non-communicable diseases (especially MAFLD). Through the collaborative efforts at these comprehensive levels, it is possible to effectively reduce the harm of air pollution in relation to MAFLD and achieve a win–win situation for the climate, the environment, and public health.

## 9. Conclusions

In conclusion, future research should focus on the long-term effects of air pollution on different populations, covering more common air pollutants besides the usual particulate matter and gases (such as emerging chemical mixtures, ultrafine particles, and indoor biomass combustion products). Greater attention should be given to susceptible populations, such as children, the elderly, pregnant women, and individuals with underlying metabolic diseases (including MAFLD). Moreover, longitudinal studies are urgently needed, particularly those combining repeated individual exposure monitoring, multi-omics methods, and detailed phenotypic analysis of liver and metabolic outcomes to clarify causal pathways and identify key susceptibility windows. Cross-regional comparisons, especially between high-income and low-income countries with different pollution characteristics, will help distinguish the interaction effects of air pollution, lifestyle factors, and socio-economic determinants. Finally, future research should not only focus on quantifying risks but also assessing the synergistic benefits of specific emission reduction strategies (such as the transition to clean energy, urban greening, and the construction of active transportation infrastructure) on health, thereby providing actionable evidence for policymakers. Only through such comprehensive, multi-level, and population-oriented integrated research can we transform scientific knowledge into effective preventive measures and global health improvement strategies.

## Figures and Tables

**Table 1:** The effects of various pollutants on MAFLD.

Year	Author	Country/ Region	Study Subject	Air Pollutant(s)	Exposure Assessment Method	Major Effects	Reference
2022	Sun, S. et al.	Taiwan	Humans	PM_2.5_	Global geographically weighted regression-adjusted satellite-derived PM_2.5_ exposure assessment	PM_2.5_ concentrations above 23.5 μg/m^3^ were positively associated with MAFLD	[41]
2024	Aimuzi, R. et al.	United Kingdom	Humans	PM_2.5_ and NO_2_	Land-use regression models	Increased risk of MAFLD through perturbating inflammation and immunity responses	[42]
2024	Bo, Y. et al.	Taiwan and Hong Kong	Humans	PM_2.5_ and NO_2_	Spatio-temporal model based on satellite data	Long-term exposure associated with higher prevalence of MAFLD and MAFLD-related advanced fibrosis	[43]
2025	Ran, S. et al.	United Kingdom	Humans	PM_2.5_, PM_10_, NO_2_ and NO_x_	Ambient monitoring data and grid-based bilinear interpolation	Air pollution-related metabolic signatures increased MAFLD development	[9]
2025	Patterson, W.B. et al.	Southern California	Humans	NO_2_ and O_3_	Inverse distance weighting interpolation from monitoring stations	Individuals with *PNPLA3*-I148M genotype more susceptible to adverse effects of air pollution	[44]
2025	Chen, Y.C. et al.	Taiwan	Humans	PM_2.5_, PM_10_, NO_2_ and NO_x_	A hybrid Kriging land-use regression model	Air pollution was correlated with increased risk of MAFLD and cirrhosis	[16]
2026	Di Ciaula, A et al.	Southern Italy (Bari)	Humans	PM_10_	Geocoded residential address-based ambient monitoring assessment	PM_10_ is an independent predictor of liver fat over-storage	[45]
2026	Zhang, Y. et al.	China	Humans	NO_2_	ChinaHighAirPollutants database-based Space-Time Extra-Trees model	NO_2_ exposure is positively associated with MAFLD prevalence among older adults	[46]
2026	Chang, M. et al.	China	Human liver specimens	PM_2.5_	Real ambient PM_2.5_ whole-body inhalation exposure model	RNF15-ASK1 axis is closely linked with PM_2.5_ exposure, accelerated metabolic damage, perturbed liver metabolic homeostasis, and steatosis, and its dysfunction promotes the steatohepatitis pathological process	[47]
2019	Ding, D. et al.	China	HepG2 cells	PM_2.5_	In vitro exposure to liposoluble extracts of collected ambient PM_2.5_	miR-26a-CD36 pathway mediates PM_2.5_-induced lipid accumulation in hepatocytes	[48]
2021	Ogino, N. et al.	Japan	HepG2 and Huh7 cells	PM_2.5_	In vitro exposure to collected ambient PM_2.5_ particle suspension	Airborne PM_2.5_ in Japan induced lipid synthesis in cultured hepatocytes, induced endoplasmic reticulum stress, and impaired autophagic degradation	[49]
2020	Li, R. et al.	China	Mice	PM_2.5_	Real ambient PM_2.5_ whole-body inhalation exposure model	Induced hepatic lipid metabolism abnormality	[50]
2023	Hu, R. et al.	China	Mice	PM_2.5_	Concentrated ambient PM_2.5_ whole-body inhalation exposure using Zhejiang Whole-body Exposure System	Dysregulated hepatic phospholipid metabolism	[51]
2023	Schneider, L.J. et al.	United States (Texas)	Mice	PM components	Controlled whole-body inhalation exposure model	Exacerbated lipid accumulation and hepatocyte injury	[52]
2023	Zhang, C. et al.	China	Mice	PM_2.5_	Experimental exposure to extract from environmental PM_2.5_ samples	Induced lipid dysfunction and hepatic steatosis	[53]
2023	Gu, W. et al.	China	Mice	PM_2.5_	Concentrated ambient PM_2.5_ whole-body inhalation exposure using Zhejiang Whole-body Exposure System	Activation of β3-AR alleviated PM_2.5_-induced hepatic lipid metabolism disturbances	[54]
2024	Xiao, Y. et al.	China	Mice	PM_2.5_	PM_2.5_ from the National Urban Air Quality Real-time Publishing Platform of China	Down-regulation of Par3 mediated PM_2.5_-induced liver injury (hepatic steatosis, inflammation, and fibrosis)	[55]
2025	Feng, M. et al.	Australia	Mice	PM_2.5_	Experimental exposure to methanol-extracted traffic-derived PM_2.5_	PM_2.5_ induced lipid accumulation in the liver	[56]

ASK1, apoptosis signal-regulating kinase 1; β3-AR, beta-3 adrenergic receptor; MAFLD, metabolic dysfunction-associated fatty liver disease; NO_x_, nitrogen oxides; RNF15, RING-finger-protein 15; PNPLA3, patatin-like phospholipase domain-containing 3; O_3_, ozone; PM, particulate matter.

## Data Availability

No new data were created or analyzed in this study. Data sharing is not applicable to this article.

## Abbreviations

The following abbreviations are used in this manuscript:

CO	Carbon monoxide
CCR2	CC-chemokine receptor 2
HCC	Hepatocellular carcinoma
HSC	Hepatic stellate cells
MAFLD	Metabolic dysfunction-associated fatty liver disease
MASH	Metabolic dysfunction-associated steatohepatitis
NAFLD	Non-alcoholic fatty liver disease
NO_x_	Nitrogen oxides
NO_2_	Nitrogen dioxide
O_3_	Ozone
PM	Particulate matter
PM_2.5_	Particulate matter < 2.5 μm
PM_10_	Particulate matter < 10 μm
PPAR	Peroxisome proliferator-activated receptor
PNPLA3	Patatin-like phospholipase domain-containing protein 3
ROS	Reactive oxygen species
TGF-β	Transforming growth factor-beta

## References

[1] Dietrich P., Hellerbrand C. (2014). Non-alcoholic fatty liver disease, obesity and the metabolic syndrome. Best Pract. Res. Clin. Gastroenterol..

[2] Tilg H., Effenberger M. (2020). From NAFLD to MAFLD: When pathophysiology succeeds. Nat. Rev. Gastroenterol. Hepatol..

[3] Fazel Y., Koenig A.B., Sayiner M., Goodman Z.D., Younossi Z.M. (2016). Epidemiology and natural history of non-alcoholic fatty liver disease. Metabolism.

[4] Younossi Z.M., Koenig A.B., Abdelatif D., Fazel Y., Henry L., Wymer M. (2016). Global epidemiology of nonalcoholic fatty liver disease-Meta-analytic assessment of prevalence, incidence, and outcomes. Hepatology.

[5] Moretti V., Romeo S., Valenti L. (2024). The contribution of genetics and epigenetics to MAFLD susceptibility. Hepatol. Int..

[6] Ilyas F., Ali H., Patel P., Sarfraz S., Basuli D., Giammarino A., Satapathy S.K. (2023). Increasing nonalcoholic fatty liver disease-related mortality rates in the United States from 1999 to 2022. Hepatol. Commun..

[7] Jonas W., Schurmann A. (2021). Genetic and epigenetic factors determining NAFLD risk. Mol. Metab..

[8] Aron-Wisnewsky J., Vigliotti C., Witjes J., Le P., Holleboom A.G., Verheij J., Nieuwdorp M., Clement K. (2020). Gut microbiota and human NAFLD: Disentangling microbial signatures from metabolic disorders. Nat. Rev. Gastroenterol. Hepatol..

[9] Ran S., Zhang J., Tian F., Qian Z.M., Wei S., Wang Y., Chen G., Zhang J., Arnold L.D., McMillin S.E. (2025). Association of metabolic signatures of air pollution with MASLD: Observational and Mendelian randomization study. J. Hepatol..

[10] GBD 2023 Disease and Injury and Risk Factor Collaborators (2025). Burden of 375 diseases and injuries, risk-attributable burden of 88 risk factors, and healthy life expectancy in 204 countries and territories, including 660 subnational locations, 1990–2023: A systematic analysis for the Global Burden of Disease Study 2023. Lancet.

[11] Orellano P., Reynoso J., Quaranta N., Bardach A., Ciapponi A. (2020). Short-term exposure to particulate matter (PM_10_ and PM_2.5_), nitrogen dioxide (NO_2_), and ozone (O_3_) and all-cause and cause-specific mortality: Systematic review and meta-analysis. Environ. Int..

[12] Muzurovic E., Mikhailidis D.P., Mantzoros C. (2021). Non-alcoholic fatty liver disease, insulin resistance, metabolic syndrome and their association with vascular risk. Metabolism.

[13] Xu M.X., Ge C.X., Qin Y.T., Gu T.T., Lou D.S., Li Q., Hu L.F., Feng J., Huang P., Tan J. (2019). Prolonged PM_2.5_ exposure elevates risk of oxidative stress-driven nonalcoholic fatty liver disease by triggering increase of dyslipidemia. Free Radic. Biol. Med..

[14] Ding R., Huang L., Yan K., Sun Z., Duan J. (2024). New insight into air pollution-related cardiovascular disease: An adverse outcome pathway framework of PM_2.5_-associated vascular calcification. Cardiovasc. Res..

[15] Chen P., Ning X., Feng W., Li Y., Chen G., Shi X., Pan Y., Shi X., Xiao Y., Liu Y. (2024). Chronic Exposure to Bioaerosols in PM_2.5_ from Garbage Stations Accelerates Vascular Aging via the NF-kappaB/NLRP3 Pathway. Adv. Sci..

[16] Chen Y.C., Pan S.C., Chin W.S., Wu C.D., Guo Y.L. (2025). Long-term exposure to ambient air pollution and the incidence of nonalcoholic fatty liver disease: A cohort study. Int. J. Epidemiol..

[17] Zhang X., Hai P., Xue J., Cai Q., Zhang J., Zhang J., Zhang D., Tang Y., Bo Y., Lyu Q. (2026). Combined effect of biological age and fine particulate matter pollution with risk of nonalcoholic fatty liver disease in the UK Biobank: A prospective cohort study. Am. J. Epidemiol..

[18] Zhu Z., Zhu W., Zhou H., Wu Z. (2025). Association between exposure to air pollutants and NAFLD/MAFLD: A meta-analysis. BMC Public Health.

[19] Kwon H.S., Ryu M.H., Carlsten C. (2020). Ultrafine particles: Unique physicochemical properties relevant to health and disease. Exp. Mol. Med..

[20] Cohen A.J., Brauer M., Burnett R., Anderson H.R., Frostad J., Estep K., Balakrishnan K., Brunekreef B., Dandona L., Dandona R. (2017). Estimates and 25-year trends of the global burden of disease attributable to ambient air pollution: An analysis of data from the Global Burden of Diseases Study 2015. Lancet.

[21] Fiordelisi A., Piscitelli P., Trimarco B., Coscioni E., Iaccarino G., Sorriento D. (2017). The mechanisms of air pollution and particulate matter in cardiovascular diseases. Heart Fail. Rev..

[22] Chen Y., Zhao C., Zhang Y., Lin Y., Shen G., Wang N., Jia X., Yang Y. (2024). Associations of ambient particulate matter and household fuel use with chronic liver disease in China: A nationwide analysis. Environ. Int..

[23] Alexeeff S.E., Deosaransingh K., Liao N.S., Van Den Eeden S.K., Schwartz J., Sidney S. (2021). Particulate Matter and Cardiovascular Risk in Adults with Chronic Obstructive Pulmonary Disease. Am. J. Respir. Crit. Care Med..

[24] Marchini T., Zirlik A., Wolf D. (2020). Pathogenic Role of Air Pollution Particulate Matter in Cardiometabolic Disease: Evidence from Mice and Humans. Antioxid. Redox Signal.

[25] Wang K., Shen D., Dai P., Li C. (2023). Particulate matter in poultry house on poultry respiratory disease: A systematic review. Poult. Sci..

[26] Sun S., Yang Q., Zhou Q., Cao W., Yu S., Zhan S., Sun F. (2022). Long-term exposure to fine particulate matter and non-alcoholic fatty liver disease: A prospective cohort study. Gut.

[27] Zhou M., Wang H., Zeng X., Yin P., Zhu J., Chen W., Li X., Wang L., Wang L., Liu Y. (2019). Mortality, morbidity, and risk factors in China and its provinces, 1990–2017: A systematic analysis for the Global Burden of Disease Study 2017. Lancet.

[28] Hopper C.P., Zambrana P.N., Goebel U., Wollborn J. (2021). A brief history of carbon monoxide and its therapeutic origins. Nitric Oxide.

[29] Chen K., Breitner S., Wolf K., Stafoggia M., Sera F., Vicedo-Cabrera A.M., Guo Y., Tong S., Lavigne E., Matus P. (2021). Ambient carbon monoxide and daily mortality: A global time-series study in 337 cities. Lancet Planet. Health.

[30] Feng C., Yang B., Wang Z., Zhang J., Fu Y., Yu B., Dong S., Ma H., Liu H., Zeng H. (2024). Relationship of long-term exposure to air pollutant mixture with metabolic-associated fatty liver disease and subtypes: A retrospective cohort study of the employed population of Southwest China. Environ. Int..

[31] Zhang Z., Jiang J., Lu B., Meng X., Herrmann H., Chen J., Li X. (2022). Attributing Increases in Ozone to Accelerated Oxidation of Volatile Organic Compounds at Reduced Nitrogen Oxides Concentrations. PNAS Nexus.

[32] GBD 2019 Risk Factors Collaborators (2020). Global burden of 87 risk factors in 204 countries and territories, 1990-2019: A systematic analysis for the Global Burden of Disease Study 2019. Lancet.

[33] Wiegman C.H., Li F., Ryffel B., Togbe D., Chung K.F. (2020). Oxidative Stress in Ozone-Induced Chronic Lung Inflammation and Emphysema: A Facet of Chronic Obstructive Pulmonary Disease. Front. Immunol..

[34] Zhang J.J., Wei Y., Fang Z. (2019). Ozone Pollution: A Major Health Hazard Worldwide. Front. Immunol..

[35] Pye H.O.T., Appel K.W., Seltzer K.M., Ward-Caviness C.K., Murphy B.N. (2022). Human-health impacts of controlling secondary air pollution precursors. Environ. Sci. Technol. Lett..

[36] Yang S., Li M., Guo C., Requia W.J., Sakhvidi M.J.Z., Lin K., Zhu Q., Chen Z., Cao P., Yang L. (2025). Associations of long-term exposure to nitrogen oxides with all-cause and cause-specific mortality. Nat. Commun..

[37] Aimuzi R., Xie Z., Qu Y., Jiang Y. (2024). Air pollution, life’s essential 8, and risk of severe non-alcoholic fatty liver disease among individuals with type 2 diabetes. BMC Public Health.

[38] Zhang X., Yang X., Hu L., Tan L., Li X., Chai Y., Ru S. (2025). Association between air pollution and risk of non-alcoholic fatty liver disease: An updated meta-analysis. Front. Public Health.

[39] Simkhovich B.Z., Kleinman M.T., Kloner R.A. (2008). Air pollution and cardiovascular injury epidemiology, toxicology, and mechanisms. J. Am. Coll. Cardiol..

[40] Ya P., Xu H., Ma Y., Fang M., Yan X., Zhou J., Li F. (2018). Liver injury induced in Balb/c mice by PM_2.5_ exposure and its alleviation by compound essential oils. Biomed. Pharmacother..

[41] Sun S., Yang Q., Zhou Q., Cao W., Yu S., Zhan S., Sun F. (2022). Long-term exposure to air pollution, habitual physical activity and risk of non-alcoholic fatty liver disease: A prospective cohort study. Ecotoxicol. Environ. Saf..

[42] Aimuzi R., Xie Z., Qu Y., Luo K., Jiang Y. (2024). Proteomic signatures of ambient air pollution and risk of non-alcoholic fatty liver disease: A prospective cohort study in the UK Biobank. Sci. Total Environ..

[43] Bo Y., Lin C., Guo C., Wong M., Huang B., Lau A., Huang Y., Lao X.Q. (2024). Chronic exposure to ambient air pollution and the risk of non-alcoholic fatty liver disease: A cross-sectional study in Taiwan and Hong Kong. Ecotoxicol. Environ. Saf..

[44] Patterson W.B., Young N.D., Holzhausen E.A., Lurmann F., Liang D., Walker D.I., Jones D.P., Liao J., Chen Z., Conti D.V. (2025). Oxidative gaseous air pollutant exposure interacts with PNPLA3-I148M genotype to influence liver fat fraction and multi-omics profiles in young adults. Environ. Pollut..

[45] Di Ciaula A., Krawczyk M., Weber S.N., Khalil M., JohnBritto J.S., Abdallah H., Portincasa P. (2026). Association between long-term exposure to PM_10_ and metabolic dysfunction-associated steatotic liver disease (MASLD) in subjects with disturbed metabolic homeostasis and genotypes at risk. Eur. J. Intern. Med..

[46] Zhang Y., Liu X., Hu Y., Cheng X., Liu W., Tian Z., Zhang Y., Chen G., Hu B., Liang C. (2026). Association of nitrogen dioxide with non-alcoholic fatty liver disease in Chinese rural older adults: The mediating role of fasting plasma glucose. Environ. Res..

[47] Chang M., Zhao J., Fan H., Shi Z., Xu M., Ge C., Zhu L., Tan J., Meng D. (2026). Metabolic insults-initialised nonalcoholic steatohepatitis promoted by fine particulate matter challenge: A mechanism involving RNF15-driven ASK1 degradation. Free Radic. Biol. Med..

[48] Ding D., Ye G., Lin Y., Lu Y., Zhang H., Zhang X., Hong Z., Huang Q., Chi Y., Chen J. (2019). MicroRNA-26a-CD36 signaling pathway: Pivotal role in lipid accumulation in hepatocytes induced by PM_2.5_ liposoluble extracts. Environ. Pollut..

[49] Ogino N., Miyagawa K., Nagaoka K., Sumida K., Kusanaga M., Oe S., Honma Y., Shibata M., Harada M., Suganuma N. (2021). Airborne fine particulate matter in Japan induces lipid synthesis and inhibits autophagy in HepG2 cells. Int. J. Biochem. Cell Biol..

[50] Li R., Wang Y., Chen R., Gu W., Zhang L., Gu J., Wang Z., Liu Y., Sun Q., Zhang K. (2020). Ambient fine particulate matter disrupts hepatic circadian oscillation and lipid metabolism in a mouse model. Environ. Pollut..

[51] Hu R., Zhang L., Qin L., Ding H., Li R., Gu W., Chen R., Zhang Y., Rajagoplan S., Zhang K. (2023). Airborne PM_2.5_ pollution: A double-edged sword modulating hepatic lipid metabolism in middle-aged male mice. Environ. Pollut..

[52] Schneider L.J., Santiago I., Johnson B., Stanley A.H., Penaredondo B., Lund A.K. (2023). Histological features of non-alcoholic fatty liver disease revealed in response to mixed vehicle emission exposure and consumption of a high-fat diet in wildtype C57Bl/6 male mice. Ecotoxicol. Environ. Saf..

[53] Zhang C., Ma T., Liu C., Ma D., Wang J., Liu M., Ran J., Wang X., Deng X. (2023). PM_2.5_ induced liver lipid metabolic disorders in C57BL/6J mice. Front. Endocrinol..

[54] Gu W., Wang R., Chai Y., Zhang L., Chen R., Li R., Pan J., Zhu J., Sun Q., Liu C. (2023). beta3 adrenergic receptor activation alleviated PM_2.5_-induced hepatic lipid deposition in mice. Sci. Total Environ..

[55] Xiao Y., Hu J., Chen R., Xu Y., Pan B., Gao Y., Deng Y., Li W., Kan H., Chen S. (2024). Impact of fine particulate matter on liver injury: Evidence from human, mice and cells. J. Hazard. Mater..

[56] Feng M., Padula M.P., Asaad S.A., Bai X., Cranfield C., Town S.E., Saad S., Oliver B.G., George J., Chen H. (2025). Prolonged exposure to low-dose traffic-derived PM_2.5_ causes fatty liver disorder in mice. J. Environ. Sci..

[57] Wang M.W., Sun L., Wen W., Wang J., Wang C.Y., Ni J., Jiang J.J., Feng Z.H., Cheng Y.R. (2022). Explore the Relationship Between Short-Term Ambient Air Pollution Exposure and Daily Outpatient Visits for Metabolic Related Fatty Liver. Risk Manag. Healthc. Policy.

[58] Guo B., Guo Y., Nima Q., Feng Y., Wang Z., Lu R., Baimayangji, Ma Y., Zhou J., Xu H. (2022). Exposure to air pollution is associated with an increased risk of metabolic dysfunction-associated fatty liver disease. J. Hepatol..

[59] Zhang Z., Guo C., Chang L.Y., Bo Y., Lin C., Tam T., Hoek G., Wong M.C., Chan T.C., Lau A.K. (2019). Long-term exposure to ambient fine particulate matter and liver enzymes in adults: A cross-sectional study in Taiwan. Occup. Environ. Med..

[60] Kim H.J., Min J.Y., Seo Y.S., Min K.B. (2019). Association of Ambient Air Pollution with Increased Liver Enzymes in Korean Adults. Int. J. Environ. Res. Public Health.

[61] Li R., Wang Y., Hou B., Lam S.M., Zhang W., Chen R., Shui G., Sun Q., Qiang G., Liu C. (2020). Lipidomics insight into chronic exposure to ambient air pollution in mice. Environ. Pollut..

[62] Patterson W.B., Holzhausen E., Chalifour B., Goodrich J., Costello E., Lurmann F., Conti D.V., Chen Z., Chatzi L., Alderete T.L. (2023). Exposure to ambient air pollutants, serum miRNA networks, lipid metabolism, and non-alcoholic fatty liver disease in young adults. Ecotoxicol. Environ. Saf..

[63] Li F.R., Liao J., Zhu B., Li X., Cheng Z., Jin C., Mo C., Wu X., Li Q., Liang F. (2023). Long-term exposure to air pollution and incident non-alcoholic fatty liver disease and cirrhosis: A cohort study. Liver Int..

[64] Brunekreef B., Strak M., Chen J., Andersen Z.J., Atkinson R., Bauwelinck M., Bellander T., Boutron M.C., Brandt J., Carey I. (2021). Mortality and Morbidity Effects of Long-Term Exposure to Low-Level PM_2.5_, BC, NO_2_, and O_3_: An Analysis of European Cohorts in the ELAPSE Project. Res. Rep. Health Eff. Inst..

[65] He X., Zhang S., Bai Q., Pan M., Jiang Y., Liu W., Li W., Gong Y., Li X. (2025). Air pollution exposure and prevalence of non-alcoholic fatty liver disease and related cirrhosis: A systematic review and meta-analysis. Ecotoxicol. Environ. Saf..

[66] Lin Y.C., Shih H.S., Lai C.Y. (2022). Long-term nonlinear relationship between PM_2.5_ and ten leading causes of death. Environ. Geochem. Health.

[67] Wang Z., Yu S., Yang B., Wang P., Yang Y., Bo Y., Wang W. (2025). The mediating and moderating effect of BMI in the relationship between air pollution and nonalcoholic fatty liver disease: A prospective cohort study. Environ. Pollut..

[68] Chen L., Jia Y., Guo Y., Chen G., Ciren Z., Chen H., Duoji Z., Xu J., Yang T., Xu H. (2023). Residential greenness associated with decreased risk of metabolic- dysfunction-associated fatty liver disease: Evidence from a large population-based epidemiological study. Ecotoxicol. Environ. Saf..

[69] Chen V.L., Oliveri A., Miller M.J., Wijarnpreecha K., Du X., Chen Y., Cushing K.C., Lok A.S., Speliotes E.K. (2023). PNPLA3 Genotype and Diabetes Identify Patients With Nonalcoholic Fatty Liver Disease at High Risk of Incident Cirrhosis. Gastroenterology.

[70] Scherz-Shouval R., Elazar Z. (2011). Regulation of autophagy by ROS: Physiology and pathology. Trends Biochem. Sci..

[71] Brook R.D., Urch B., Dvonch J.T., Bard R.L., Speck M., Keeler G., Morishita M., Marsik F.J., Kamal A.S., Kaciroti N. (2009). Insights into the mechanisms and mediators of the effects of air pollution exposure on blood pressure and vascular function in healthy humans. Hypertension.

[72] Yuan C.S., Lai C.S., Tseng Y.L., Hsu P.C., Lin C.M., Cheng F.J. (2021). Repeated exposure to fine particulate matter constituents lead to liver inflammation and proliferative response in mice. Ecotoxicol. Environ. Saf..

[73] Han B., Xu J., Zhang Y., Li P., Li K., Zhang N., Han J., Gao S., Wang X., Geng C. (2022). Associations of Exposure to Fine Particulate Matter Mass and Constituents with Systemic Inflammation: A Cross-Sectional Study of Urban Older Adults in China. Environ. Sci. Technol..

[74] Becker S., Momoh J., Biancacci I., Mockel D., Wang Q., May J.N., Su H., Candels L.S., Berres M.L., Kiessling F. (2023). Intermittent Fasting Primes the Tumor Microenvironment and Improves Nanomedicine Delivery in Hepatocellular Carcinoma. Small.

[75] Xu X., Liu C., Xu Z., Tzan K., Zhong M., Wang A., Lippmann M., Chen L.C., Rajagopalan S., Sun Q. (2011). Long-term exposure to ambient fine particulate pollution induces insulin resistance and mitochondrial alteration in adipose tissue. Toxicol. Sci..

[76] Zheng Z., Xu X., Zhang X., Wang A., Zhang C., Huttemann M., Grossman L.I., Chen L.C., Rajagopalan S., Sun Q. (2013). Exposure to ambient particulate matter induces a NASH-like phenotype and impairs hepatic glucose metabolism in an animal model. J. Hepatol..

[77] Du Z., Lin L., Li Y., Sun M., Liang Q., Sun Z., Duan J. (2022). Combined exposure to PM_2.5_ and high-fat diet facilitates the hepatic lipid metabolism disorders via ROS/miR-155/PPARgamma pathway. Free Radic. Biol. Med..

[78] Tian L., Gao S., Zeng Y., Lin L., Li T., Sun Z., Yu Y. (2026). PM_2.5_ exacerbate nonalcoholic fatty liver disease through activating hepatocytes TXNIP/NLRP3/FoxO1 signaling pathway in ob/ob mice. Free Radic. Biol. Med..

[79] Han C.Y., Rho H.S., Kim A., Kim T.H., Jang K., Jun D.W., Kim J.W., Kim B., Kim S.G. (2018). FXR Inhibits Endoplasmic Reticulum Stress-Induced NLRP3 Inflammasome in Hepatocytes and Ameliorates Liver Injury. Cell Rep..

[80] Zheng Z., Zhang X., Wang J., Dandekar A., Kim H., Qiu Y., Xu X., Cui Y., Wang A., Chen L.C. (2015). Exposure to fine airborne particulate matters induces hepatic fibrosis in murine models. J. Hepatol..

[81] Wang M., Tan J., Zhou J., Yi B., Huang Z. (2020). Farnesoid X receptor mediates hepatic steatosis induced by PM_2.5_. Environ. Sci. Pollut. Res. Int..

[82] Frampton M.W., Boscia J., Roberts N.J., Azadniv M., Torres A., Cox C., Morrow P.E., Nichols J., Chalupa D., Frasier L.M. (2002). Nitrogen dioxide exposure: Effects on airway and blood cells. Am. J. Physiol. Lung Cell. Mol. Physiol..

[83] Tan H.H., Fiel M.I., Sun Q., Guo J., Gordon R.E., Chen L.C., Friedman S.L., Odin J.A., Allina J. (2009). Kupffer cell activation by ambient air particulate matter exposure may exacerbate non-alcoholic fatty liver disease. J. Immunotoxicol..

[84] Hasegawa Y., Okamura T., Nakajima H., Kitagawa N., Majima S., Okada H., Senmaru T., Ushigome E., Nakanishi N., Hamaguchi M. (2023). Metabolic outcomes and changes in innate immunity induced by diesel exhaust particles airway exposure and high-fat high-sucrose diet. Life Sci..

[85] Parker R., Weston C.J., Miao Z., Corbett C., Armstrong M.J., Ertl L., Ebsworth K., Walters M.J., Baumart T., Newland D. (2018). CC chemokine receptor 2 promotes recruitment of myeloid cells associated with insulin resistance in nonalcoholic fatty liver disease. Am. J. Physiol. Gastrointest. Liver Physiol..

[86] Liu C., Xu X., Bai Y., Wang T.Y., Rao X., Wang A., Sun L., Ying Z., Gushchina L., Maiseyeu A. (2014). Air pollution-mediated susceptibility to inflammation and insulin resistance: Influence of CCR2 pathways in mice. Environ. Health Perspect..

[87] Romeo S., Kozlitina J., Xing C., Pertsemlidis A., Cox D., Pennacchio L.A., Boerwinkle E., Cohen J.C., Hobbs H.H. (2008). Genetic variation in PNPLA3 confers susceptibility to nonalcoholic fatty liver disease. Nat. Genet..

[88] Bruschi F.V., Claudel T., Tardelli M., Caligiuri A., Stulnig T.M., Marra F., Trauner M. (2017). The PNPLA3 I148M variant modulates the fibrogenic phenotype of human hepatic stellate cells. Hepatology.

[89] Breton C.V., Song A.Y., Xiao J., Kim S.J., Mehta H.H., Wan J., Yen K., Sioutas C., Lurmann F., Xue S. (2019). Effects of air pollution on mitochondrial function, mitochondrial DNA methylation, and mitochondrial peptide expression. Mitochondrion.

[90] Hou L., Zhang X., Dioni L., Barretta F., Dou C., Zheng Y., Hoxha M., Bertazzi P.A., Schwartz J., Wu S. (2013). Inhalable particulate matter and mitochondrial DNA copy number in highly exposed individuals in Beijing, China: A repeated-measure study. Part. Fibre Toxicol..

[91] Zhou B., Liu C., Xu L., Yuan Y., Zhao J., Zhao W., Chen Y., Qiu J., Meng M., Zheng Y. (2021). N^6^-Methyladenosine Reader Protein YT521-B Homology Domain-Containing 2 Suppresses Liver Steatosis by Regulation of mRNA Stability of Lipogenic Genes. Hepatology.

[92] Yan Z., Zhang Y., Nan N., Ji S., Lan S., Qin G., Sang N. (2024). YTHDC2 mediated RNA m^6^A modification contributes to PM_2.5_-induced hepatic steatosis. J. Hazard. Mater..

[93] Qiu Y.N., Wang G.H., Zhou F., Hao J.J., Tian L., Guan L.F., Geng X.K., Ding Y.C., Wu H.W., Zhang K.Z. (2019). PM_2.5_ induces liver fibrosis via triggering ROS-mediated mitophagy. Ecotoxicol. Environ. Saf..

[94] Rautenberg E.K., Hamzaoui Y., Coletta D.K. (2022). Mini-review: Mitochondrial DNA methylation in type 2 diabetes and obesity. Front. Endocrinol..

[95] Pirola C.J., Gianotti T.F., Burgueno A.L., Rey-Funes M., Loidl C.F., Mallardi P., Martino J.S., Castano G.O., Sookoian S. (2013). Epigenetic modification of liver mitochondrial DNA is associated with histological severity of nonalcoholic fatty liver disease. Gut.

[96] Byun H.M., Panni T., Motta V., Hou L., Nordio F., Apostoli P., Bertazzi P.A., Baccarelli A.A. (2013). Effects of airborne pollutants on mitochondrial DNA methylation. Part. Fibre Toxicol..

[97] Sookoian S., Flichman D., Scian R., Rohr C., Dopazo H., Gianotti T.F., Martino J.S., Castano G.O., Pirola C.J. (2016). Mitochondrial genome architecture in non-alcoholic fatty liver disease. J. Pathol..

[98] Sunny N.E., Parks E.J., Browning J.D., Burgess S.C. (2011). Excessive hepatic mitochondrial TCA cycle and gluconeogenesis in humans with nonalcoholic fatty liver disease. Cell Metab..

[99] Ngan H.L., Zhang J., Chen Y., Song Y., Qi Z., Yang Z., Yan H., Cai Z. (2026). Hepatotoxicity Prediction and Multi-omics Reveal Mitochondrial and Lipid Metabolic Dysregulation in PM_2.5_-Induced Liver Fibrosis. Environ. Health.

[100] Jang T.Y., Ho C.C., Liang P.C., Wu C.D., Wei Y.J., Tsai P.C., Hsu P.Y., Hsieh M.Y., Lin Y.H., Hsieh M.H. (2024). Air pollution associate with advanced hepatic fibrosis among patients with chronic liver disease. Kaohsiung J. Med. Sci..

[101] Chen Y.S., Lin H.Y., Lin C.T., Huang S.H., Yeh M.L., Yu M.L., Lee J.C., Chung Y.C., Li W.M., Li C.C. (2026). Chronic, Environmentally Relevant PM_2.5_ Exposure Exacerbates Metabolic Dysfunction-Associated Steatotic Liver Disease and Early-Stage Renal Dysfunction in a Rodent Model. Kaohsiung J. Med. Sci..

[102] Markevych I., Wolf K., Hampel R., Breitner S., Schneider A., von Klot S., Cyrys J., Heinrich J., Doring A., Beelen R. (2013). Air pollution and liver enzymes. Epidemiology.

[103] Barouki R., Samson M., Blanc E.B., Colombo M., Zucman-Rossi J., Lazaridis K.N., Miller G.W., Coumoul X. (2023). The exposome and liver disease—How environmental factors affect liver health. J. Hepatol..

[104] Zhang J., Ran S., Wei S., Tian F., Chen L., Yang Z., Chen G., Lin H. (2025). Associations of MAFLD subtypes and air pollutants with multi-system morbidity and all-cause mortality: A prospective cohort study. Ecotoxicol. Environ. Saf..

[105] Lan Y., Zhang H., Wu A., Huang J., Guo Z., Chen Y. (2025). Long-term exposure to air pollution and liver fibrosis in the elderly with MASLD. Sci. Rep..

[106] Kleiner D.E., Makhlouf H.R. (2016). Histology of Nonalcoholic Fatty Liver Disease and Nonalcoholic Steatohepatitis in Adults and Children. Clin. Liver Dis..

[107] Juanola O., Martinez-Lopez S., Frances R., Gomez-Hurtado I. (2021). Non-Alcoholic Fatty Liver Disease: Metabolic, Genetic, Epigenetic and Environmental Risk Factors. Int. J. Environ. Res. Public Health.

[108] Kim S., Lee A.Y., Cho M.H. (2021). Inhaled exposure to air fresheners aggravated liver injury in a murine model of nonalcoholic fatty acid liver disease. Heliyon.

[109] Wang Z.J., Yu H., Hao J.J., Peng Y., Yin T.T., Qiu Y.N. (2021). PM_2.5_ promotes Drp1-mediated mitophagy to induce hepatic stellate cell activation and hepatic fibrosis via regulating miR-411. Exp. Cell Res..

[110] Song Y., Wei J., Li R., Fu R., Han P., Wang H., Zhang G., Li S., Chen S., Liu Z. (2023). Tyrosine kinase receptor B attenuates liver fibrosis by inhibiting TGF-beta/SMAD signaling. Hepatology.

[111] Pritchett N., Spangler E.C., Gray G.M., Livinski A.A., Sampson J.N., Dawsey S.M., Jones R.R. (2022). Exposure to Outdoor Particulate Matter Air Pollution and Risk of Gastrointestinal Cancers in Adults: A Systematic Review and Meta-Analysis of Epidemiologic Evidence. Environ. Health Perspect..

[112] So R., Chen J., Mehta A.J., Liu S., Strak M., Wolf K., Hvidtfeldt U.A., Rodopoulou S., Stafoggia M., Klompmaker J.O. (2021). Long-term exposure to air pollution and liver cancer incidence in six European cohorts. Int. J. Cancer.

[113] Pedersen M., Andersen Z.J., Stafoggia M., Weinmayr G., Galassi C., Sorensen M., Eriksen K.T., Tjonneland A., Loft S., Jaensch A. (2017). Ambient air pollution and primary liver cancer incidence in four European cohorts within the ESCAPE project. Environ. Res..

[114] Ha S., Wong V.W., Zhang X., Yu J. (2024). Interplay between gut microbiome, host genetic and epigenetic modifications in MASLD and MASLD-related hepatocellular carcinoma. Gut.

[115] Iaccarino J., Mignini I., Maresca R., Giansanti G., Esposto G., Borriello R., Galasso L., Ainora M.E., Gasbarrini A., Zocco M.A. (2025). The Impact of Air Pollution on the Lung-Gut-Liver Axis: Oxidative Stress and Its Role in Liver Disease. Antioxidants.

[116] Cho S.H., Hwang S.E., Kim H.J., Kim S., Kang Y.H., Yun J.M., Park J.H. (2025). Association between long-term exposure to ambient air pollution and an increased risk of steatotic liver disease. Sci. Rep..

[117] Sun Q., Yue P., Deiuliis J.A., Lumeng C.N., Kampfrath T., Mikolaj M.B., Cai Y., Ostrowski M.C., Lu B., Parthasarathy S. (2009). Ambient air pollution exaggerates adipose inflammation and insulin resistance in a mouse model of diet-induced obesity. Circulation.

[118] Miller D.B., Karoly E.D., Jones J.C., Ward W.O., Vallanat B.D., Andrews D.L., Schladweiler M.C., Snow S.J., Bass V.L., Richards J.E. (2015). Inhaled ozone (O3)-induces changes in serum metabolomic and liver transcriptomic profiles in rats. Toxicol. Appl. Pharmacol..

[119] Zhang Y., Shi J., Ma Y., Yu N., Zheng P., Chen Z., Wang T., Jia G. (2023). Association between Air Pollution and Lipid Profiles. Toxics.

[120] Li R., Sun Q., Lam S.M., Chen R., Zhu J., Gu W., Zhang L., Tian H., Zhang K., Chen L.C. (2020). Sex-dependent effects of ambient PM_2.5_ pollution on insulin sensitivity and hepatic lipid metabolism in mice. Part. Fibre Toxicol..

[121] Li K., Zhang Q., Wang T., Rong R., Hu X., Zhang Y. (2022). Laboratory investigation of pollutant emissions and PM_2.5_ toxicity of underground coal fires. Sci. Total Environ..

[122] Feldstein A.E., Alkhouri N., De Vito R., Alisi A., Lopez R., Nobili V. (2013). Serum cytokeratin-18 fragment levels are useful biomarkers for nonalcoholic steatohepatitis in children. Am. J. Gastroenterol..

[123] Hsieh S., Leaderer B.P., Feldstein A.E., Santoro N., McKay L.A., Caprio S., McConnell R. (2018). Traffic-related air pollution associations with cytokeratin-18, a marker of hepatocellular apoptosis, in an overweight and obese paediatric population. Pediatr. Obes..

[124] Lu T.Y., Wu C.D., Huang Y.T., Chen Y.C., Chen C.J., Yang H.I., Pan W.C. (2024). Exposure to PM_2.5_ Metal Constituents and Liver Cancer Risk in REVEAL-HBV. J. Epidemiol..

[125] Chang S.T., Chou Y.H., Wu C.D., Nfor O.N., Lu W.Y., Huang C.N., Liaw Y.P. (2026). Synergistic Effect of Ambient PM_2.5_ Exposure and Waist-Hip Ratio on Non-Alcoholic Fatty Liver Disease Risk in a Taiwanese Population. Int. J. Med. Sci..

[126] Kelishadi R., Poursafa P. (2011). Obesity and air pollution: Global risk factors for pediatric non-alcoholic fatty liver disease. Hepat. Mon..

[127] Lin C., Rountree C.B., Methratta S., LaRusso S., Kunselman A.R., Spanier A.J. (2014). Secondhand tobacco exposure is associated with nonalcoholic fatty liver disease in children. Environ. Res..

[128] Vilar-Gomez E., Nephew L.D., Vuppalanchi R., Gawrieh S., Mladenovic A., Pike F., Samala N., Chalasani N. (2022). High-quality diet, physical activity, and college education are associated with low risk of NAFLD among the US population. Hepatology.

[129] Kong X., Huang R., Geng R., Wu J., Li J., Wu Y., Zhao Y., You D., Yu H., Du M. (2024). Associations of ambient air pollution and lifestyle with the risk of NAFLD: A population-based cohort study. BMC Public Health.

[130] Hiza H.A., Casavale K.O., Guenther P.M., Davis C.A. (2013). Diet quality of Americans differs by age, sex, race/ethnicity, income, and education level. J. Acad. Nutr. Diet..

[131] Liu J., Clark L.P., Bechle M.J., Hajat A., Kim S.Y., Robinson A.L., Sheppard L., Szpiro A.A., Marshall J.D. (2021). Disparities in Air Pollution Exposure in the United States by Race/Ethnicity and Income, 1990–2010. Environ. Health Perspect..

[132] Jbaily A., Zhou X., Liu J., Lee T.H., Kamareddine L., Verguet S., Dominici F. (2022). Air pollution exposure disparities across US population and income groups. Nature.

[133] Guo H., Li W., Yao F., Wu J., Zhou X., Yue Y., Yeh A.G.O. (2020). Who are more exposed to PM_2.5_ pollution: A mobile phone data approach. Environ. Int..

[134] Zhang J., Mauzerall D.L., Zhu T., Liang S., Ezzati M., Remais J.V. (2010). Environmental health in China: Progress towards clean air and safe water. Lancet.

[135] Kang N., Li P., Xue T., Zhu T. (2024). Development of a Method to Determine the Environmental Burden of Diseases and an Application to Identify Factors Driving Changes in the Number of PM_2.5_-Related Deaths in China between 2000 and 2010. Environ. Health.

[136] Wang Y., Wang Y., Xu H., Zhao Y., Marshall J.D. (2022). Ambient Air Pollution and Socioeconomic Status in China. Environ. Health Perspect..

[137] Hu W., Liu Z., Hao H.R., Yu W.N., Wang X.Q., Shao X.J., Wu X.J., Wen S.R., Fan Y.Q., Ni Y.J. (2020). Correlation between income and non-alcoholic fatty liver disease in a Chinese population. Ann. Endocrinol..

[138] Huangfu G., Chan D.C., Pang J., Jaltotage B., Watts G.F., Lan N.S.R., Bell D.A., Ihdayhid A.R., Ayonrinde O.T., Dwivedi G. (2025). Triglyceride to High-Density Lipoprotein Cholesterol Ratio as a Marker of Subclinical Coronary Atherosclerosis and Hepatic Steatosis in Familial Hypercholesterolemia. Endocr. Pract..

[139] Fuller C.H., Feeser K.R., Sarnat J.A., O’Neill M.S. (2017). Air pollution, cardiovascular endpoints and susceptibility by stress and material resources: A systematic review of the evidence. Environ. Health.

[140] Wierzbicka A., Omelekhina Y., Saber A.T., Bloom E., Gren L., Poulsen S.S., Strandberg B., Pagels J., Jacobsen N.R. (2022). Indoor PM_2.5_ from occupied residences in Sweden caused higher inflammation in mice compared to outdoor PM_2.5_. Indoor Air.

[141] Nasr P., Shang Y., Wester A., Strandberg R., Widman L., Lazarus J.V., Hagstrom H. (2024). Socioeconomic factors associated with the presence of and outcomes in metabolic dysfunction-associated steatotic liver disease. Liver Int..

[142] Wang X., Guo B., Yang X., Li J., Baima Y., Yin J., Yu J., Xu H., Zeng C., Feng S. (2022). Role of Liver Enzymes in the Relationship Between Particulate Matter Exposure and Diabetes Risk: A Longitudinal Cohort Study. J. Clin. Endocrinol. Metab..

[143] Romanello M., Walawender M., Hsu S.C., Moskeland A., Palmeiro-Silva Y., Scamman D., Ali Z., Ameli N., Angelova D., Ayeb-Karlsson S. (2024). The 2024 report of the Lancet Countdown on health and climate change: Facing record-breaking threats from delayed action. Lancet.

[144] Liu M., Yang S., Ye Z., Zhang Y., He P., Zhou C., Zhang Y., Qin X. (2023). Residential green and blue spaces with nonalcoholic fatty liver disease incidence: Mediating effect of air pollutants. Ecotoxicol. Environ. Saf..

